# *Eurotium Cristatum* Postfermentation of Fireweed and Apple Tree Leaf Herbal Teas

**DOI:** 10.1155/2021/6691428

**Published:** 2021-09-23

**Authors:** Tatiana A. Efimenko, Elena F. Shanenko, Tatiana G. Mukhamedzhanova, Olga V. Efremenkova, Yuriy A. Nikolayev, Elena N. Bilanenko, Marina V. Gernet, Artem G. Grishin, Ivan N. Serykh, Sergey V. Shevelev, Byazilya F. Vasilyeva, Svetlana N. Filippova, Galina I. El-Registan

**Affiliations:** ^1^Gause Institute of New Antibiotics, Moscow 119021, Russia; ^2^Moscow State University of Food Production, Moscow 125080, Russia; ^3^Research Center of Biotechnology RAS, Winogradsky Institute of Microbiology, Moscow 117312, Russia; ^4^Lomonosov Moscow State University, Faculty of Biology, Moscow 119991, Russia; ^5^V.M. Gorbatov Federal Research Center of Food Systems RAS, All-Russian Research Institute of Brewing, Nonalcoholic and Wine Industry, Moscow 109316, Russia; ^6^LLC “Sistema”, Moscow 115230, Russia; ^7^Company “MOYCHAY.RU”, Moscow 101000, Russia

## Abstract

Fungi *Eurotium* spp. are the main biological agents that ferment the leaves of the *Camellia sinensis* tea bush to form a popular food product, postfermented tea. The fungus *E. cristatum*, stored in the collection of the Gause Institute of New Antibiotics under the number INA 01267, was isolated and identified from a briquette of Fujian Chinese tea. The species identification was carried out based on morphocultural characteristics and DNA sequencing. This study is aimed at determining the feasibility of making postfermented herbal teas using *E. cristatum* and to evaluate their quality. Autofermented herbal teas from *Chamaenerion angustifolium* (fireweed) and *Malus domestica* (apple tree) served as the starting material for this study. The change in the concentration of phenolic compounds, organic acids, sugars, and free amino acids was observed for herbal teas subjected to postfermentation with *E. cristatum* INA 01267. It was found that the *E. cristatum* INA 01267 strain does not have antimicrobial activity and does not form mycotoxins, which is an indicator of food safety.

## 1. Introduction

It is generally accepted that tea is one of the most popular drinks in the world today, and China is recognized as a major tea producer and exporter. In the classic version, tea is obtained from the processed leaves of the tea bush *Camellia sinensis* or *C. assamica*. The properties of tea largely depend on the processes of enzymatic oxidation of the leaves of the tea bush. All teas are divided into fermented (or autofermented) and postfermented teas. Autofermented teas are produced by biochemical processes in plant cells. Depending on the degree of oxidation, autofermented teas are divided into green teas, which are fermented for a short time, and black teas, which are fermented for several weeks. Postfermented teas, extra to autofermentation, are additionally fermented by microorganisms; therefore, they are the most enriched with biologically active substances, since they contain not only the products of tea components autofermentation, but also the products of their transformation by microorganisms, as well as microbial metabolites [1–3]. During postfermentation in wet conditions, such popular teas and tea drinks as Kombucha drink of Chinese origin and traditional Japanese Ishizuchi-kurocha tea are obtained. In the presence of water, during natural postfermentation, the bacteria of the family *Acetobacteriaceae* (mainly of the genus *Komagataeibacter* spp. for Kombucha) and *Lactobacillaceae* (namely, *Lactiplantibacillus plantarum* and *L. pentosus* for Ishizuchi-kurocha) predominate. In addition, but in significantly smaller quantities, there are bacteria of the families *Paenibacillaceae*, *Staphylococcaceae*, *Streptococcaceae*, *Lachnospiraceae*, *Bacteroidaceae*, and *Bifidobacteriaceae*. About 30 species of fungi have been described, but yeasts of the *Saccharomycetaceae* (with a predominance *Zygosaccharomyces* spp. or *Candida* spp.), *Schizosaccharomycetaceae* (*Schizosaccharomyces* spp.), and *Pichiaceae* (*Brettanomyces* spp.) families prevail [4–9].

During postfermentation of dry plant mass, xerophytic fungi predominate, mainly ascomycetes of the genera *Aspergillus*, *Penicillium*, *Saccharomyces*, and *Eurotium* (*Aspergillus* anamorph). One of the most popular postfermented teas in China is black brick tea named “Fujian” tea or “Fu Tea” (Fujian Province Guang Fu Tea Co., Ltd., China). The study of the microbial community of Fujian postfermented tea revealed mainly fungi of the genera *Eurotium* (more than 15 species, primarily *E. cristatum*) [2, 10]. The presence of golden-brown spots of this fungus on tea briquettes, called “golden flowers,” is an indicator of the high tea quality [10–12].

During postfermentation, the biochemical composition of tea changes. Currently, about 2000 biologically active compounds found in teas have been shown. Among them, there are catechins, organic acids, flavonols, theaflavins, teagallins, alkaloids, sugars, amino acids, vitamins, etc. [3]. Such a large number of active compounds explain the rich variety of teas, differences in their organoleptic properties, and effect on the organism. Postfermented teas are not only popular drinks, but also have medicinal properties: they normalize lipid metabolism, and they have antimicrobial, antidiabetic, antioxidant, and antimutagenic effects [10, 13–15]. In addition to useful substances, fungi form undesirable or even harmful substances from the point of food microbiology view. Special attention is paid to fungi, which are associated not only with the fact that they affect the composition and give products useful properties, but also with the ability of many of them to produce antibiotics or toxins that cause serious food poisoning. However, on plant food with low water activity (*a*_*w*_ = 0.6–0.0), nontoxigenic strains of fungi, including those from the genus *Eurotium*, develop effectively. The best studied are the isolates of *E. cristatum* that develop on dry leaves of *C. sinensis*. These xerophytic fungi are an important factor in the formation of tea properties and are actively used in industrial technologies in China for the production of postfermented teas [2, 10, 16].

In addition to teas made from the leaves of *C. sinensis*, there are other drinks produced from leaves and herbs of various plants. These drinks are called herbal teas. The most studied of this group are teas made from mate (*Ilex paraguariensis*), hibiscus (*Hibiscus sabdariffa*), mint (*Mentha piperita*), and chamomile (*Matricaria recutita*). These drinks are used for medicinal purposes as well as to quench thirst and pleasure. Most of these plants are characterized by a fairly high content of phenolic compounds, antioxidants, and other compounds that affect the psychoemotional state [17].

**Figure 1:**
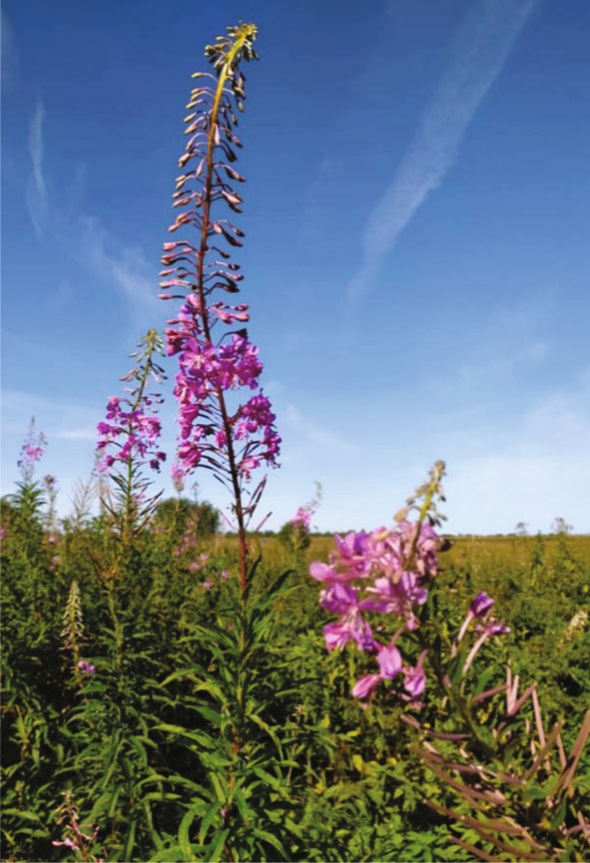
*Chamaenerion angustifolium* (fireweed).

In Russia, along with the culture of consumption of traditional tea from *C. sinensis* leaves, herbal teas have been popular since ancient times [18, 19]. The most popular is the one made from *Chamaenerion angustifolium* (Figure 1). It is a perennial herb with narrow leaves that resemble willow leaves. It is widespread in the northern hemisphere, often found in forest clearings and burned-out areas. Hence, the names of this plant are fireweed, firetop, great willowherb, and rosebay willowherb. *Ch. angustifolium* in Russia has many names depending on the region, but the most common is Ivan-tea. In addition to fireweed tea, drinks with good organoleptic characteristics and high antioxidant activity are obtained from other plants, in particular, from the leaves of fruit and berry crops [19]. Technologies for microbial postfermentation of herbal teas have not been developed in Russia, and they are not industrially produced.

This study is generally aimed at examining the possibility of using *E. cristatum* for postfermentation of two herbal teas. The experimental data in this publication represent the first step towards the development of technology to produce postfermented herbal teas in Russia. The specific objectives of this study were as follows: to isolate a strain of *E. cristatum* from a Fujian postfermented tea briquette and describe it; to analyze its growth on fermented herbal teas from *Chamaenerion angustifolium* (fireweed) and *Malus domestica* (apple tree); to determine the composition of phenolic compounds, sugars, organic acids, and free amino acids in fermented and postfermented teas; to compare the compositions of two herbal teas with each other and with the literature data; to determine the potential antimicrobial activity on the panel of test strains; and to establish the absence/presence of fungal mycotoxins.

## 2. Materials and Methods

### 2.1. Sample Collection

The fungus was isolated from postfermented black brick tea named either “Fujian” tea or “Fu Tea” (Fujian Province Guang Fu Tea Co., Ltd., Hunan Province, China). Upon visual inspection of the Fujian postfermented tea briquettes, yellow patches of fungus growth were clearly visible. From these places, the material was taken to prepare the suspension. When sowing a suspension of briquetted tea on agar media, the growth of fungal colonies of the same morphological type was observed. The microbiological purity of the isolates was confirmed by at least five consecutive passages on various agar media.

### 2.2. Cultural Media and Cultural Conditions for the Fungus *E. Cristatum* INA 01267

The isolated fungus was incubated on autofermented herbal teas from the plants *Ch. angustifolium* (fireweed) and *M. domestica* (apple tree leaves) (MOYCHAY.RU, Moscow, Russia).

Four agar media were used (%): (1) Czapek medium: sucrose—3, NaNO_3_—0.3, KH_2_PO_4_—0.1, MgSO_4_ • 7H_2_О—0.05, KCl—0.05, FeSO_4_ • 7H_2_O—0.001, and agar—1.5; tap water; (2) Sabouraud agar medium: glucose—4, peptone—0.7, soy bean flour—0.3, yeast extract—0.4, and agar—1.5, tap water; (3) modified agar medium #2 Gause: glucose—1, peptone—0.5, tryptone—0.3, NaCl—0.5, and agar—2, tap water, pH 7.2–7.4; and (4) mineral agar medium #1 Gause: soluble starch—2, K_2_HPO_4_—0.05, MgSO_4_ • 7H_2_О—0.05, KNO_3_—0.1, NaCl—0.05, FeSO_4_ • 7H_2_O—0.001, and agar—2, distillate water, pH 7.2–7.4.

The fungus was incubated at 28°C.

Medium 1 was used for storage and maintenance of fungus; the duration of cultivation was 7 days. Media 1-4 were used for morphological study; the duration of cultivation was 2-21 days.

Media 1-3 without agar were used for submerged cultivation. Submerged cultivation was carried out in 750 mL Erlenmeyer flasks with 150 mL of medium on a rotary shaker (200 rpm).

### 2.3. Fungus Identification

The species of the isolated strain was determined based on morphological and cultural characteristics, as well as analysis of gene sequences.

Morphological and cultural features of the fungus were considered with growth on different media over 2-21 days. The preparations were examined with 150x, 600x, and 1500x magnifications using a Mikmed-6 light microscope (LOMO-Microanalysis, Russia). The nucleotide sequences of ribosomal RNA genes and some other regions were also identified (Table 1).

Extraction of total DNA from fungal biomass was performed using DNeasy PowerSoil Kit (Qiagen Inc., USA). Polymerase chain reaction (PCR) was carried out using a set of PCR Master Mix reagents (Thermo Scientific, USA). The final volume of the 50 *μ*L PCR mix included 25 *μ*L 2X PCR Master Mix, 0.5 *μ*mol of each of the primers, 1-100 ng of isolated DNA and nuclease-free water. Fungal primer sets and thermocycling programs are described in Table 1. PCR was performed on a Thermal Cycler 2720 device (Applied Biosystems, USA).

The amplification products were purified by DNA reprecipitation under mild conditions using 0.125 M ammonium acetate in 70% aqueous ethanol and visualized on a 1% agarose gel (using TBE Tris-borate buffer) at an electric field strength of 7.6 V/cm. The same PCR primers for sequencing regions ITS-D1/D2, LSU, and RPB2 and only forward primers (EF-595F, *β*t2a, and gpd1) for regions TEF1-*α*, *β*-tub, and GAPDH were used. The nucleotide sequences were determined by the Sanger method on automatic sequencer Genetic Analyzer 3500 (Applied Biosystems, USA). The DNAStar Lasergene SeqMan v.7.1.0 (DNASTAR Inc., Madison, WI, USA) and Mega 7 [20] were used to view, edit, and align the obtained raw sequence data. Reference nucleotide sequences were obtained from GenBank databases [21] and CBS [22].

### 2.4. Determination of Biochemical Parameters of Herbal Teas as a Result of Postfermentation with *E. Cristatum* INA 01267

Into a 500 mL Erlenmeyer flask, 20 g of autofermented herbal teas was added, sterilized by autoclaving (1 atm, 40 min) and inoculated with an aqueous suspension, containing ascospores and mycelium fragments to a concentration of 10^3^ CFU/g of tea. The humidity of the environment was 20%. Postfermentation of herbal teas with the fungus *E. cristatum* INA 01267 was carried out stationary for 14 days at 28°C. The obtained samples of postfermented biomass were dried at 50°C to a moisture content of 5%. Aqueous extracts were prepared from the obtained dried biomass, for which 1 g of biomass was added to 20 mL of water and extracted with stirring for 20 min at 80°C, followed by centrifugation at 3000 × g for 15 min. The following compounds were determined in aqueous extracts by HPLC: phenolic compounds and organic acids were determined on an Agilent 1200 chromatograph (Agilent Technologies, USA) with a diode array detector (DAD), a Hypersit ODS C18 250 × 4.6 mm 5 *μ*m chromatographic column (Thermo Fisher Scientific, USA); the solvent system was a phosphate buffer with pH 2.5 with acetonitrile in the ratio 13 : 87 [23, 24]; sugars were determined on an Agilent 1200 chromatograph (Agilent Technologies, USA) with a refractive index *detector* (RID), chromatographic column Luna NH2 100A 250 × 4.6 mm 5 *μ*m (Phenomenex, USA), the solvent system was a mixture of acetonitrile with water in a ratio of 75 : 25, and temperature of the mobile phase was 40°C [23, 24]; free amino acids were determined on an Agilent 1200 chromatograph (Agilent Technologies, USA) with a diode array detector (DAD), a chromatographic column Luna C18 (2) 150 × 4.4 mm 5 *μ*m (Phenomenex, USA) with a guard column; the solvent system was 0.1 M sodium acetate pH 6.4-acetonitrile in a ratio of 9 : 1 [23, 25].

After analyzing the extracts, the content of compounds was recalculated and expressed in micrograms per gram of tea dry weight of (*μ*g/g d.w.).

### 2.5. Elucidation of the Possibility of Mycotoxin Formation by *E. Cristatum* INA 01267

The *E. cristatum* INA 01267 strain was grown under submerged cultivation on medium 1 for 7 days. The grown culture was centrifuged at 5000 × g for 20 min.

The presence of mycotoxins in the culture broth supernatant was determined by HPLC on a TermoFinnigan chromatograph with a diode array detector, chromatographic column Hypersil™ BDS C18 200 × 4.6 mm 5 *μ*m (Thermo Fisher Scientific, USA); the solvent systems were as follows: for aflatoxin B1, toluene-ethyl acetate-formic acid in a volume ratio 80 : 40 : 95; for patulin and zearalenone, buffer solution of potassium dihydrogen phosphate pH 3.0-acetonitrile in a volume ratio of 95 : 5; and for deoxynivalenol, methanol-water in a volume ratio of 95 : 5 [23, 26].

HPLC detection limits for mycotoxins were 0.003 mg/kg for aflatoxin, 0.2 mg/kg for deoxynivalenol, 0.2 mg/kg for zearalenone, and 0.01 mg/kg for patulin.

### 2.6. *E. Cristatum* INA 01267: Determination of the Possibility to Produce Antimicrobial Substances

To determine the antimicrobial activity, the *E. cristatum*INA 01267 was cultured under submersion conditions in the described above media 1-3. The samples of the culture liquid were taken on the 2, 4, 7, 10, 14, and 21 days of growth. The presence of antibiotic activity in the culture liquid was determined by diffusion into agar medium. For this, the studied samples of the culture liquid were introduced into the wells of Petri dishes with agar medium 3 infected with the test strain. After incubation, the level of antimicrobial activity was judged by presence or absence of zones of growth inhibition of test cultures around the wells. Discs with some antibacterial and antifungal antibiotics served as control (levofloxacin (LVX5), novobiocin (NB5), chloramphenicol (C30), amikacin (AN30), cefotaxime (CTX30), and ciprofloxacin (CIP5); Becton, Dickinson and Company, USA).

The following microorganisms were used as test strains to determine the antibiotic activity of *E. cristatum* INA 01267: Gram-positive bacteria *Bacillus subtilis* АТСС 6633, *B. pumilus* NCTC 8241, *B. mycoides* 537, *Micrococcus luteus* NCTC 8340, *Leuconostoc mesenteroides* VKPM B-4177, *Staphylococcus aureus* FDA 209P, and *S. aureus* INA 00761, as well as mycobacteria *Mycobacterium smegmatis* VKPM Ac 1339 and *M. smegmatis* mc^2^ 155; Gram-negative bacteria *Escherichia coli* ATCC 25922, *Pseudomonas aeruginosa* ATCC 27853, and *Comamonas terrigena* VKPM B-7571; and fungi *Aspergillus niger* INA 00760 and *Saccharomyces cerevisiae* RIA 259.

*L. mesenteroides* VKPM B-4177, *A. niger* INA 00760, and *Sac. cerevisiae* RIA 259 were incubated at 28°C, and the rest of the test strains were at 37°C.

### 2.7. Data Processing

The data were analyzed using MS Excel 2016 and presented as mean ± SD of three replicates.

## 3. Results

### 3.1. Fungus Identification

**Figure 2:**
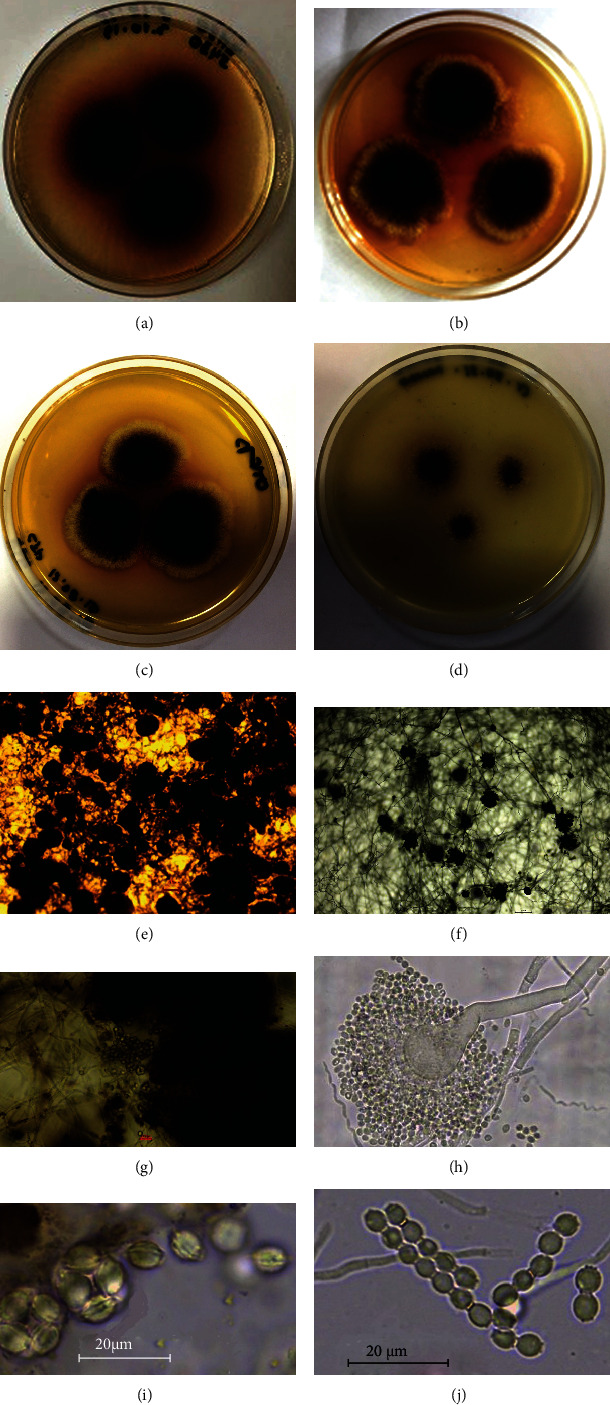
Seven-day fungal growth on media (a) Czapek, (b) Sabouraud, (c) modified agar medium #2 Gause, and (d) mineral agar medium #1 Gause; (e) cleistothecia on Sabouraud medium after 4 days of growth; (f) conidiophores on Sabouraud medium with 40% of glucose after 21 days of growth, upper part of the colony; (g) destroyed cleistothecia and asci; (h) conidiophore with single-row phialides covering the conidiophore's head and chains of conidia; (i) asci and ascospores; (j) chains of conidia separated by disjunctors.

The strain grew approximately equally well on organic media Czapek, Sabouraud, and modified agar medium #2 Gause, but grew poorly on mineral agar medium #1 Gause (Figures 2(a)–2(d)). On Czapek's medium, the fungus had an olive-beige, later olive-brown colony coloration, and a characteristic red-brown exopigment coloration (Figure 2(a)); the growth rate was 5.2 mm/day. On the fourth day of growth, large amounts of cleistothecia formed on all agar media (Figures 2(e) and 2(g)). The sizes of cleistothecia were from 45 to 108 *μ*m in diameter. Each ascospore contained 8 ascospores with a pronounced equatorial crest and a prickly uneven surface; the spore size was 3.6 × 4.5 *μ*m (Figures 2(g) and 2(i)). Conidial sporulation was observed only when grown on Czapek's medium with 40% glucose. Conidiophores with spores were located in the upper layer of colonies above the mycelium with cleistothecia (Figure 2(f)). The heads of the conidiophores were covered with one layer of phialides, from which the chains of conidia extend. Conidia were spherical to elliptical in shape, smooth surface, and 2.6 to 2.9 *μ*m in diameter along the long axis (Figures 2(h) and 2(j)). By the totality of the described characteristics, the fungi corresponded to the description of the species *Eurotium cristatum* (Raper et Fennell) Malloch et Cain, teleomorph, or *Aspergillus cristatus* Raper et Fennell, anamorph [27, 28].

To confirm the identified species, the sequences of six DNA regions were determined (Table 2). In four cases out of six (for genes ITS-D1/D2, RPB2, TEF1-*α*, and *β*-tub), the coincidence is 99.67-100% with the sequences of *Aspergillus cristatus* genes in the database, which confirmed the species affiliation of the strain identified by morphological-cultural characteristics. The fungus strain was deposited in the collection of the Gause Institute of New Antibiotics as *Eurotium cristatum* (anamorph of *Aspergillus cristatus*) INA 01267.

### 3.2. Changes in Biochemical Parameters as a Result of Postfermentation with *E. Cristatum* INA 01267

The transformation of initial plant materials by the fungus *E. cristatum* INA 01267 was judged by the change in the content of phenolic compounds, organic acids, sugars, and amino acids in the extracts of postfermented herbal teas.

Comparative analysis of the composition of herbal teas showed that the total content of phenolic compounds as a result of postfermentation in fireweed tea was significantly higher than in the apple tree leaf tea (5626.44 and 251.12 *μ*g/g, respectively). The results showed that the postfermentation of the fireweed tea biomass led to an approximately twofold increase in the content of sinapic acid and syringaldehyde and to the complete disappearance of coniferyl and synaptic aldehydes. The postfermentation of tea biomass from the apple tree leaves led to the appearance of gallic acid in an amount of 126 *μ*g/g and to the disappearance of such components as syringaldehyde and vanillin.

**Figure 3:**
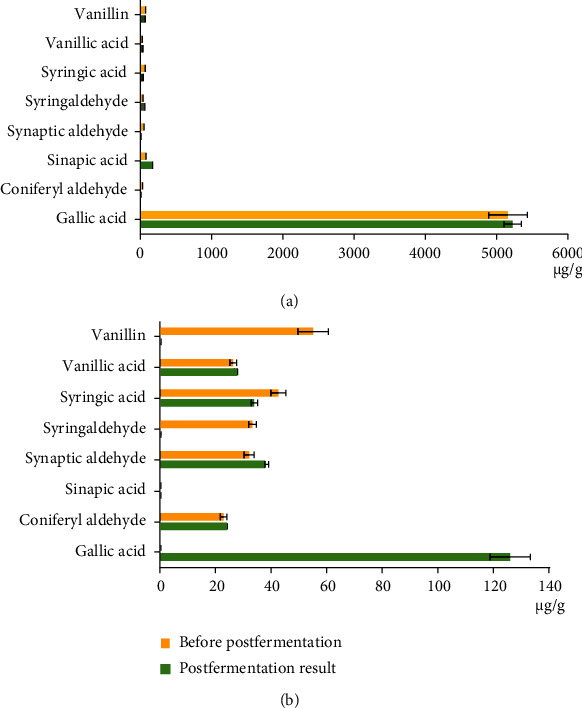
Impact of *Eurotium cristatum* INA 01267 postfermentation on the composition of phenolic compounds in herbal teas (*μ*g/g). (a) Fireweed tea. (b) Apple tree leaf tea. Presented data of phenolic compound content are mean values from 3 independent extractions ± standard deviation (SD).

It was noted that the total content of low molecular weight phenolic compounds before and after their postfermentation practically did not change in the case of the fireweed tea biomass and slightly increased (by 18%) in the case of the apple tree leaf tea, which indicated the activity of redox processes during postfermentation (Figure 3 and Table S1).

**Figure 4:**
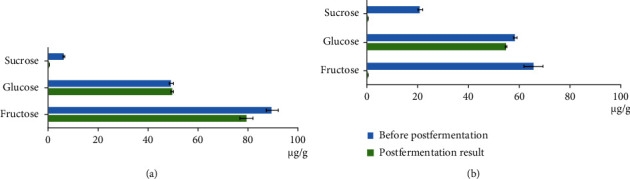
Impact of *Eurotium cristatum* INA 01267 postfermentation on the composition of sugars in herbal teas (*μ*g/g). (a) Fireweed tea. (b) Apple tree leaf tea. Presented data of sugar content are mean values from 3 independent extractions ± standard deviation (SD).

The analysis of the sugar content in the extracts of postfermented teas showed that during the postfermentation of fireweed tea, the fungus completely utilized the sucrose, while the fructose content slightly decreased (by 12%). During the postfermentation of the apple tree leaf tea, the sucrose and fructose were completely utilized. The glucose level in both cases remained practically constant (Figure 4 and Table S2).

**Figure 5:**
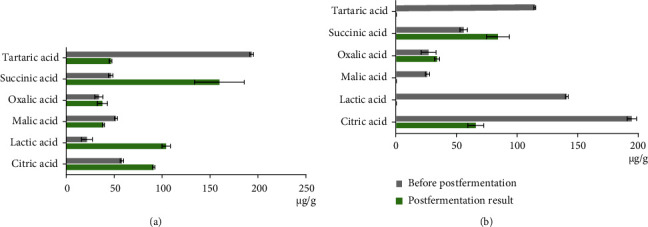
Impact of *Eurotium cristatum* INA 01267 postfermentation on the composition of organic acids in herbal teas (*μ*g/g). (a) Fireweed tea. (b) Apple tree leaf tea. The presented data of the organic acid content were mean values from 3 independent extractions ± standard deviation (SD).

After *Eurotium cristatum* INA 01267 growth, the malic and tartaric acids were utilized in both samples and in the case of apple tree leaf tea, completely. The oxalic acid content in both samples changed insignificantly. The hanging content of the succinic acid (up to 345% and 150%) was also typical for both samples. The difference between the samples consisted in a sharp change, as a result of the postfermentation, in the concentrations of citric and lactic acids: an increase in the fireweed tea sample (up to 157% and 488%, respectively) and a decrease in the apple tree leaf tea (down to 34% and 0%, respectively) (Figure 5 and Table S3).

**Figure 6:**
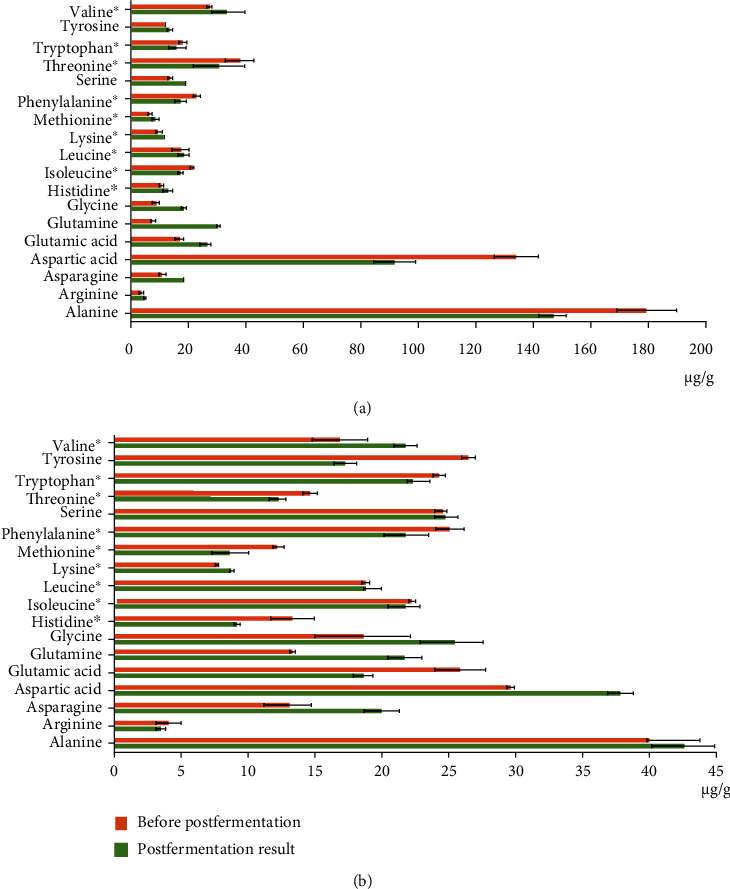
(a) Impact of *Eurotium cristatum* INA 01267 postfermentation on the composition of amino acids in herbal teas (*μ*g/g). Fireweed tea. Presented data of free amino acid content are mean values from 3 independent extractions ± standard deviation (SD). ^∗^Essential amino acids. (b) Impact of *Eurotium cristatum* INA 01267 postfermentation on the composition of amino acids in herbal teas (*μ*g/g). Apple tree leaf tea. Presented data of free amino acid content are mean values from 3 independent extractions ± standard deviation (SD). ^∗^Essential amino acids.

The analysis of free amino acids in extracts of the fireweed tea and the apple tree leaf tea before and after cultivation of *E. cristatum* INA 01267 showed that as a result of fungi growth, the content of amino acids asparagine, glutamine, glycine, and valine significantly increased in both samples (in the range of 23-305%). In addition, in the fireweed tea sample, the content of arginine, glutamic acid, methionine, and serine also increased (by 26-58%), and in the sample of the apple tree leaf tea, the content of aspartic acid increased (up to 127%). In both extracts, before and after cultivation of *E. cristatum* INA 01267, all 9 free essential amino acids were present, and the content of essential amino acids such as histidine, leucine, lysine, methionine, phenylalanine, tyrosine, and valine had increased in the fireweed tea. In the biomass of apple tree leaf tea, the content of valine significantly increased (up to 129%) and the content of glycine increased slightly (up to 114%). In both biomasses, the content of individual amino acids changed, but the total content remained practically unchanged (Figure 6 and Table S4).

### 3.3. Determination of Toxic and Antibiotic Activity of *E. Cristatum* INA 01267 under Submerged Cultivation

In biotechnological tea production, a special place is given to the safety issues, since some species of *Aspergillus* fungi during the growth can synthesize mycotoxins and antibiotics, the presence of which is harmful and undesirable for food products. The culture liquid after incubation of the *E. cristatum* INA 01267 strain in Czapek's liquid medium was analyzed by HPLC for the content of mycotoxins: aflatoxin B1, deoxynivalenol, zearalenoene, and patulin. The results showed that *E. cristatum* INA 01267 did not form these mycotoxins. The analysis of the antibiotic activity of the culture liquid of the *E. cristatum* INA 01267 strain at the age of 2 to 21 days against 12 test strains of bacteria and fungi also showed no activity.

## 4. Discussion

In accordance with the aim of this study—to create technology of postfermented Russian herbal teas production, we isolated the fungus from postfermented black brick tea Fujian. The isolated strain was identified as *E. cristatum* and deposited in the Gause Institute collection as INA 01267. It confirms the previously published data that *E. cristatum* is most often found in Fujian tea briquette. The ability of *E. cristatum*, for which the main substrates are soil and plant leaves, to develop not only on the leaves of the *C. sinensis* tea bush, but also on other plant material, was quite obvious and confirmed in our studies for two autofermented herbal teas commercially produced in Russia.

Like all fungi, *E. cristatum* INA 01267 has a pronounced hydrolase activity, which ensures its growth on plant material due to low molecular weight compounds formed from plant biopolymers. We analyzed the content of low molecular weight carbohydrates (phenolic compounds, organic acids, and sugars) and free amino acids in the herbal tea extracts before and after *E. cristatum* INA 01267 growth.

The effect of microbial fermentation (postfermentation) on the composition of phenolic compounds has been well studied for the leaves of the tea bush *C. sinensis*. The oxidation of gallocatechin gallate (GCG) and epigalocatechin gallate (EGCG) contained in tea leaves leads to the formation of their condensation products—theaflavins and theorubigins. When fermented by fungi of the genus *Aspergillus*, carried out in the technology of Pu-erh tea, the content of complex phenolic compounds, such as EGCG and GCG, decreases and the amount of both simple catechins not associated with gallic acid, free gallic acid, and its conversion products increases [29].

It was previously established that tannins (enotein) predominated in the composition of phenolic compounds of fireweed herb, whereas flavonoids (quercetin, mericitin, quercitrin, kaempferol, and quercitrin-galloyl-galactoside) and phenols such as gallic acid and ellagic acid which were present in smaller quantities [19]. In the process of autofermentation, the composition of phenolic compounds underwent significant changes, and the content of phenolic acids and flavonoids, which had high antioxidant activity, increased. Particularly, the active antioxidants were the compounds having hydroxyl groups in the orthoposition, such as quercetin. The oxidized phenolic compounds could condense to form tannin or be reduced by interaction with other antioxidants present. According to our data, the similar processes occurred during the postfermentation by the *E. cristatum* INA 01267 strain of herbal teas from the fireweed and the apple tree leaves. The redox reactions led to a change in the ratio of aldehydes and acids (Figure 3 and Table S1). In the postfermented fireweed tea, the amount of syringic acid decreased (by 1.5 times) and the amount of syringaldehyde increased with a constant content of vanillin; the synaptic aldehyde disappeared completely, while the content of sinapic acid doubled; also, the coniferyl aldehyde completely disappeared. The phenolic composition and transformation of phenols in the apple tree leaves were much less studied than in the fireweed herb, but the direction of the processes was of a general nature [18].

According to our data, during the postfermentation, the concentration of gallic acid in fireweed tea increased slightly (about 1%), while in apple tree leaf tea, it increased dramatically, namely, by 550 times. However, in absolute terms, the content of gallic acid in fireweed tea is about 40 times higher than in apple tree leaf tea. The gallic acid is found in many plants, and it is part of the complex of tannins. It is an antioxidant and, like salicylic acid, suppresses the accumulation of white blood cells in extravascular areas of the tissue and inhibits chronic inflammation. It has also been shown that gallic acid affects the immune system by stimulating the formation of immunoglobulins G [30]. Accordingly, the accumulation of gallic acid in teas is a useful property. As an impact of *Eurotium cristatum* INA 01267 growth, vanillin and syringaldehyde disappeared, the amount of coniferyl and synaptic aldehydes increased slightly, and the content of syringic acid decreased slightly. These data indicated hydrolysis of complex phenols and ongoing oxidative processes (Figure 3 and Table S1). The results obtained correspond to the data on the decomposition of the complex phenols of leaves of the tea bush *Camellia* by fungi of the genus *Eurotium* [31]. According to the literature data, the number of phenolic compounds gradually decreases with prolonged fermentation. Thus, during postfermentation of teas, complex phenolic compounds are decomposed, as a result of which their decay products accumulate, which are later partially assimilated by the fungus [31].

The content of sugars (sucrose and fructose) decreased until the complete disappearance of sucrose in both teas, as well as fructose in the apple tree leaf tea and a decrease in its level in the fireweed tea. The glucose, called the “energy currency of the cell,” was maintained at a constant level (Figure 4 and Table S2).

The accumulation of succinic and oxalic acids, as well as the decrease in the content of tartaric and malic acids, was common to both teas; the difference consisted an increase in the content of lactic and citric acids in the fireweed tea and the decrease in them in the tea from apple leaves (Figure 5 and Table S3). Succinic acid, a desirable component of tea, is an antioxidant with a wide spectrum of biological activity: it increases the body's immune status, has an antitoxic effect, and improves the state of the cardiovascular system [32].

When analyzing the amino acid composition of both herbal teas, a higher content of free amino acids (including all essential amino acids) was noted, and the concentrations of which were mainly in the range of 4-38 *μ*g/g. The expectation was the dominance of aspartic acid and alanine in the fireweed tea (91.63 and 146.97 *μ*g/g, respectively), as well as a high content of threonine, valine, and glutamine, while in the apple tree leaf tea was aspartic acid and alanine (Figure 6 and Table S4). The concentration of amino acids before and after fungal fermentation did not change as much as in the case of carbohydrates (Figures 3–5 and Table S1-3).

In general, as a result of postfermentation, the fireweed tea had a higher content of phenolic compounds, sugars, organic acids, and amino acids compared to the apple tree leaf tea (Table 3). The popularity of herbal tea from fireweed in Russia has developed historically, and, apparently, it was developed based on taste and benefits, and now it is confirmed by laboratory data. In this study, we did not plan to study the composition of teas in dynamics, but we are planning such a task in order to develop production technology, optimize the composition of teas, and increase their useful properties. The composition of tea varies depending not only on the raw material, strain, but also on the duration of postfermentation. Therefore, knowing the composition of the listed substances in dynamics, it is possible to influence, for example, the sweetness or acidity of the drink.

The results obtained by us cannot be accurately compared with the published data of other authors due to different research conditions. It is possible to note a high general trend associated with the hydrolysis of high-molecular compounds on the one hand and the consumption of the obtained products by the fungus on the other. The closest experimental conditions are given in the publication Xiao et al. by amino acids.  Table 4 shows a comparison of the results obtained, although in our study, the duration of fermentation was 14 days, and in the cited work is 12 and 16 days [33].

Table 4 shows that postfermented tea from the leaves of the tea bush *C. sinensis* is about 4 times richer in free amino acids than tea from *Ch. angustifolium* (fireweed) and *M. domestica* (apple tree leaves). It is also shown that postfermentation with a duration of 12 days reduces the total content of amino acids to 38%, while postfermentation *Ch. angustifolium* and *M. domestica* decreases only by 3-4%. It once again underlines the importance of postfermentation conditions, in particular its duration.

A necessary characteristic of fungal strains for use in food industry technologies is the absence of toxicity. The safety of the *E. cristatum* INA 01267 strain was confirmed by HPLC analysis of the culture broth due to the absence of common toxins aflatoxin B1, patulin, deoxynivalenol, and zearalenone produced by toxigenic fungi [34]. It was previously found that the expression of aflatoxin biosynthesis-related genes (aflD, aflQ, and aflS) in *E. cristatum* was downregulated [35].

Many publications have noted the antimicrobial effect of postfermented teas. This activity can be explained by the composition of the tea leaf or the hydrolysis products of the components of the tea leaves under the action of *E. cristatum*, as well as by the biosynthesis products of the fungus grown in a microbiological medium without tea leaves. The antimicrobial activity of teas has been repeatedly proven against pathogenic Gram-positive and Gram-negative bacteria [36, 37]. The authors considered plant polyphenols as the main antimicrobial agents in teas, presumably affecting the activity of various enzymes and the stability of the membranes of pathogenic bacteria [38]. The proposed mechanism of the antimicrobial activity of Chinese teas is a characteristic of phenolic compounds, in particular, alkylresorcinols [39–41].

A detailed description of the antimicrobial properties of teas and their composition was presented in the literature [3, 38]. There were few proven antimicrobial products produced by *E. cristatum*. When fermented under submerged conditions, an endophytic fungus *E. cristatum* EN-220, isolated from the marine alga *Sargassum thunbergii*, produced previously unknown indole alkaloids, namely, cristatumins A-D, along with nine known congeners. Interestingly, these compounds differed in their antibiotic activity: antibacterial, insecticidal, anthelmintic, and fungicidal. However, the MIC of these compounds was high and amounted to tens and hundreds of micrograms per milliliter [11, 42]. The probiotic strain *E. cristatum* was isolated by the Chinese brick tea Fuzhuan and was tested for its *in vitro* activity against aflatoxigenic *Aspergillus flavus*. It was established by GC/MS that there were many antifungal substances present in the *E. cristatum* HNYYWX.21 culture filtrate. In addition, this strain inhibited the production of aflatoxins in *A. flavus*. The chemical structure of active compounds had not yet been established [35].

The described *E. cristatum* INA 01267 strain did not show antibacterial or antifungal activity when tested against 14 test cultures. Antimicrobial activity was absent in the culture liquid obtained during the growth of the fungus in three nutrient media of different composition. We regarded this as a desirable characteristic for the strain that can be used in the food industry for postfermentation in herbal teas.

## 5. Conclusions

The growth of *E. cristatum* INA 01267 strain was efficient on autofermented herbal teas from fireweed and apple tree leaves. As a result of postfermentation, the plant material was hydrolyzed and some useful low molecular weight metabolites, such as succinic acid, essential amino acids, and phenols, accumulated. The *E. cristatum* INA 01267 strain did not produce toxins and antimicrobial substances, which is desirable in the food industry. Postfermented tea made from apple tree leaves is somewhat inferior to tea made from fireweed (“Ivan-tea”), a traditional drink in Russia, in terms of the content of nutrients.

## Figures and Tables

**Table 1:** Studied DNA regions of the fungus and primers and PCR annealing temperature.

DNA regions/genes	Symbols	Thermocycling programs	Primers (5′→3′)	
			Symbols	Sequences
Entire ITS rDNA and fragment of 28S rDNA	ITS-D1/D2	(1) 94°C–5 min (2) 33 cycles with temperature interval 94°C–1 min, 51°C–1 min, 72°C–1 min (3) 72°C–7 min	ITS1f	TCCGTAGGTGAACTTGCG
			NL4	GGTCCGTGTTTCAAGG
Large subunit 28S of ribosomal DNA	LSU	(1) 94°C–5 min (2) 33 cycles with temperature interval 94°C–1 min, 49°C–1 min, 72°C–2 min (3) 72°C–7 min	LR0R	ACCCGCTGAACTTAAGC
			LR5	TCCTGAGGGAAACTTCG
RNA polymerase II gene	RPB2	(1) 94°C–5 min (2) 9 cycles 94°C–1 min, at 60°C to 50°C–1 min (with 1 degree decrement each cycle), 72°C–1.5 min (3) 32 cycles 94°C–1 min, 50°C–1.5 min, 72°C–1.5 min (4) 72°C–7 min	fRPB2-5F	GAYGAYMGWGATCAYTTYGG
			fRPB2-7cR	CCAT(AG)GCTTG(CT)TT(AG)CCCAT
Translation elongation factor 1-*α* gene	TEF1-*α*	(1) 94°C–5 min (2) 9 cycles 94°C–1 min, at 66°C to 56°C–1 min (with 1 degree decrement each cycle), 72°C–1.5 min (3) 32 cycles 94°C–1 min, 56°C–1.5 min, 72°C–1.5 min (4) 72°C–7 min	EF-595F	CGTGACTTCATCAAGAACATG
			EF-1567R	ACHGTRCCRATACCACCRATCTT
*β*-tubulin gene	*β*-tub	(1) 94°C–5 min (2) 9 cycles 94°C–1 min, at 64°C to 54°C–1 min (with 1 degree decrement each cycle), 72°C–1.5 min (3) 32 cycles 94°C–1 min, 54°C–1.5 min, 72°C–1.5 min (4) 72°C–7 min	*β*t2a	GGTAACCAAATCGGTGCTGCTTTC
			*β*t2b	ACCCTCAGTGTAGTGACCCTTGGC
Glycerol 3-phosphate dehydrogenase gene	GAPDH	(1) 94°C–5 min (2) 9 cycles 94°C–1 min, at 65°C to 55°C–1 min (with 1 degree decrement each cycle), 72°C–1.5 min (3) 32 cycles 94°C–1 min, 55°C–1.5 min, 72°C–1.5 min (4) 72°C–7 min	gpd1	CAACGGCTTCGGTCGCA TTG
			gpd2	GCCAAGCAGTTGGTTGTGC

**Table 2:** Alignment of gene sequences of the strain *Eurotium cristatum* (anam. *Aspergillus cristatus*) INA 01267 with DNA sequences from GenBank.

DNA regions/genes	Species	Alignment (%)	Sequence length (b.p.)	Sequence number in GenBank
ITS-D1/D2	*Aspergillus amstelodami* *Aspergillus cristatus* *Eurotium heterocaryoticum* *Aspergillus hollandicus* *Aspergillus montevidensis*	100	1079	MN966538
RPB2	*Aspergillus cristatus*	100	997	MT326207
TEF1-*α*	*Aspergillus chevalieri* *Aspergillus cristatus*	99.67	302	MT326208
	*Aspergillus niger*	99.34		
*β*-tub	*Aspergillus cristatus*	100	355	MT319133
LSU	*Aspergillus amstelodami* *Aspergillus glaucus* *Aspergillus montevidensis* *Aspergillus proliferans* *Eurotium herbariorum* *Eurotium rubrum* *Eurotium spiculosum*	100	848	MN966539
GAPDH	*Aspergillus glaucus*	94.72	437	MT326209

**Table 3:** Impact of *Eurotium cristatum* INA 01267 postfermentation on the composition of biologically active compounds from four different groups (*μ*g/g).

Total^∗^	Fireweed tea		Apple tree leaf tea	
	Before postfermentation	Postfermentation result	Before postfermentation	Postfermentation result
Phenolic compounds	5546.27	5626.44	213.33	251.12
Sugars	144.99	129.2	145.44	55.7
Organic acids	404.56	476.94	559.31	184.54
Amino acids	556.04	533.80	351.52	357.58

^∗^The sum of the total average values for the three experiments.

**Table 4:** Comparison of the effect of *Eurotium cristatum* postfermentation on the composition of amino acids in various teas.

Amino acids	Fireweed tea				Apple tree leaf tea				Pingwu Fuzhuan brick tea [33]					
	Before postfermentation		Postfermentation result		Before postfermentation		Postfermentation result		Before postfermentation		Postfermentation result		Postfermentation result	
Duration of postfermentation (day)	0		14		0		14		0		12		16	
	*μ*g/g	%	*μ*g/g	%	*μ*g/g	%	*μ*g/g	%	*μ*g/g	%	*μ*g/g	%	*μ*g/g	%
TEAAs (His, Ile, Leu, Lys, Met, Phe, Thr, Trp, Val)	170.32	100	164.76	97	155.65	100	145.62	94	335.65^a^	100	278.9^a^	83	285.6^a^	85
TNEAAs (Ala, Arg, Asn, Asp, Cys, Glu, Gln, Gly, Pro, Ser, Tyr)	385.72^b^	100	369.04^b^	96	195.87^b^	100	211.96^b^	108	1937.2^c^	100	585.05^c^	30	521.7^c^	27
Total	556.04	100	533.8	96	351.52	100	357.58	102	2272.85	100	863.95	38	807.3	36

TEAAs: total essential amino acids; TNEAAs: total nonessential amino acids; ^a^excluding Met and Ile; ^b^excluding Cys and Pro; ^c^excluding Asn and Gln.

## Data Availability

The data of Figures 3–6 used to support the findings of this study are included within the supplementary information files (Table S1-S4). The data used to support the findings of this study are included within the article.

## Supplementary Materials

Supplementary Materials Table S1: impact of *Eurotium cristatum* INA 01267 postfermentation on the composition of phenolic compounds in herbal teas (*μ*g/g). Table S2: impact of *Eurotium cristatum* INA 01267 postfermentation on the composition of sugars in herbal teas (*μ*g/g). Table S3: impact of *Eurotium cristatum* INA 01267 postfermentation on the composition of organic acids in herbal teas (*μ*g/g). Table S4: impact of *Eurotium cristatum* INA 01267 postfermentation on the composition of amino acids in herbal teas (*μ*g/g).
