# Supplementary material for: Eurotium Cristatum Postfermentation of Fireweed and Apple Tree Leaf Herbal Teas
Source: Int J Food Sci. 2021 Sep 23;2021:6691428. doi: 10.1155/2021/6691428 (PMC8500772; doi:10.1155/2021/6691428)
Supplement: Supplementary Materials — Table S1: impact of Eurotium cristatum INA 01267 postfermentation on the composition of phenolic compounds in herbal teas (μg/g). Table S2: impact of Eurotium cristatum INA 01267 postfermentation on the composition of sugars in herbal teas (μg/g). Table S3: impact of Eurotium cristatum INA 01267 postfermentation on the composition of organic acids in herbal teas (μg/g). Table S4: impact of Eurotium cristatum INA 01267 postfermentation on the composition of amino acids in herbal teas (μg/g). [file 6691428.f1.doc]

**Supplementary Materials**

Table S1: Impact of *Eurotium cristatum* INA 01267 post-fermentation on the composition of phenolic compounds in herbal teas (μg/g).

| Phenolic compounds | Fireweed tea | | Apple tree leaves tea | |
| --- | --- | --- | --- | --- |
|  | Before  post-fermentation | Post-fermentation result | Before  post-fermentation | Post-fermentation result |
| Gallic acid | 5159.00±279.50 | 5218.33±120.48 | 0.23±0.21 | 126.00±7.21 |
| Coniferyl aldehyde | 34.63±5.17 | 0.17±0.15 | 23.00±1.20 | 24.20±0.20 |
| Sinapic acid | 83.77±1.10 | 176.60±5.91 | 0.17±0.15 | 0.27±0.25 |
| Synaptic aldehyde | 52.67±1.43 | 0.23±0.21 | 32.13±1.63 | 38.53±0.60 |
| Syringaldehyde | 35.63±2.26 | 65.57±5.76 | 33.43±1.35 | 0.20±0.20 |
| Syringic acid | 75.90±3.87 | 50.98±2.52 | 42.67±2.60 | 34.17±1.12 |
| Vanillic acid | 31.27±1.33 | 40.13±0.83 | 26.50±1.2 | 27.40±0.50 |
| Vanillin | 73.40±1.97 | 74.43±3.56 | 55.20±5.44 | 0.35±0.07 |
| Total | 5546.27* | 5626.44* | 213.33* | 251.12* |

Presented data of phenolic compounds content are mean values from 3 independent extractions ± standard deviation (SD). *The sum of the average values for the three experiments.

Table S2: Impact of *Eurotium cristatum* INA 01267 post-fermentation on the composition of sugars in herbal teas (μg/g).

| Sugars | Fireweed tea | | Apple tree leaves tea | |
| --- | --- | --- | --- | --- |
|  | Before  post-fermentation | Post-fermentation result | Before  post-fermentation | Post-fermentation result |
| Fructose | 89.53±2.32 | 79.23±2.66 | 65.87±3.90 | 0.37±0.06 |
| Glucose | 49.23±0.75 | 49.60±0.40 | 58.57±0.51 | 55.00±0.20 |
| Sucrose | 6.23±0.21 | 0.37±0.06 | 21.00±0.92 | 0.33±0.06 |
| Total | 144.99* | 129.2* | 145.44* | 55.7* |

Presented data of sugars content are mean values from 3 independent extractions ± standard deviation (SD). *The sum of the average values for the three experiments.

Table S3: Impact of *Eurotium cristatum* INA 01267 post-fermentation on the composition of organic acids in herbal teas (μg/g).

| Organic acids | Fireweed tea | | | | Apple tree leaves tea | | | |
| --- | --- | --- | --- | --- | --- | --- | --- | --- |
|  | Before  post-fermentation | | Post-fermentation result | | Before  post-fermentation | | Post-fermentation result | |
|  | μg/g | % | μg/g | % | μg/g | % | μg/g | % |
| Citric acid | 58.00±1.73 | 100 | 90.90±1.04 | 157 | 194.50±3.85 | 100 | 65.93±6.60 | 34 |
| Lactic acid | 21.33±5.52 | 100 | 104.07±4.15 | 488 | 140.97±1.06 | 100 | 0.23±0.21 | 0 |
| Malic acid | 51.70±1.35 | 100 | 38.67±0.58 | 75 | 25.97±1.63 | 100 | 0.27±0.25 | 1 |
| Oxalic acid | 34.03±3.99 | 100 | 37.50±5.41 | 110 | 27.07±6.02 | 100 | 33.87±1.75 | 125 |
| Succinic acid | 46.27±2.00 | 100 | 159.53±25.79 | 345 | 56.00±3.1 | 100 | 83.97±9.17 | 150 |
| Tartaric acid | 193.23±1.66 | 100 | 46.27±1.36 | 24 | 114.80±0.72 | 100 | 0.27±0.31 | 0 |
| Total | 404.56* | 100 | 476.94* | 118 | 559.31* | 100 | 184.54* | 33 |

Presented data of organic acids content are mean values from 3 independent extractions ± standard deviation (SD). *The sum of the average values for the three experiments.

Table S4: Impact of *Eurotium cristatum* INA 01267 post-fermentation on the composition of amino acids in herbal teas (µg/g).

| Amino acids | Fireweed tea | | | | Apple tree leaves tea | | | |
| --- | --- | --- | --- | --- | --- | --- | --- | --- |
|  | Before  post-fermentation | | Post-fermentation result | | Before  post-fermentation | | Post-fermentation result | |
|  | µg/g | % | µg/g | % | µg/g | % | µg/g | % |
| Alanine | 179.23±10.45 | 100 | 146.97±4.32 | 82 | 40.10±3.75 | 100 | 42.60±2.31 | 106 |
| Arginine | 3.46±0.50 | 100 | 4.36±0.33 | 126 | 4.01±0.91 | 100 | 3.49±0.30 | 87 |
| Asparagine | 10.82±1.05 | 100 | 18.03±0.06 | 167 | 13.05±1.73 | 100 | 20.00±1.25 | 153 |
| Aspartic acid | 134.17±7.54 | 100 | 91.63±6.86 | 68 | 29.70±0.26 | 100 | 37.83±0.95 | 127 |
| Glutamic acid | 16.76±1.27 | 100 | 26.40±1.31 | 158 | 25.93±1.81 | 100 | 18.79±0.61 | 72 |
| Glutamine | 7.49±0.63 | 100 | 30.37±0.38 | 405 | 13.31±0.26 | 100 | 21.70±1.18 | 163 |
| Glycine | 8.74±0.80 | 100 | 18.57±0.40 | 212 | 18.65±3.51 | 100 | 25.43±2.12 | 136 |
| Histidine* | 10.67±0.28 | 100 | 12.79±1.37 | 120 | 13.37±1.56 | 100 | 9.21±0.16 | 69 |
| Isoleucine* | 21.17±0.38 | 100 | 17.23±0.45 | 81 | 22.17±0.21 | 100 | 21.67±1.17 | 98 |
| Leucine* | 17.35±2.60 | 100 | 18.31±1.73 | 106 | 18.86±0.23 | 100 | 18.96±0.98 | 101 |
| Lysine* | 9.18±1.49 | 100 | 11.35±0.07 | 124 | 7.85±0.05 | 100 | 8.93±0.13 | 114 |
| Methionine* | 6.51±0.47 | 100 | 8.41±0.99 | 129 | 12.26±0.31 | 100 | 8.66±1.39 | 71 |
| Phenylalanine* | 22.77±1.07 | 100 | 17.09±1.92 | 75 | 25.13±0.99 | 100 | 21.87±1.61 | 87 |
| Serine | 13.74±0.53 | 100 | 19.21±0.07 | 140 | 24.60±0.20 | 100 | 24.80±0.87 | 101 |
| Threonine* | 37.83±4.77 | 100 | 30.60±8.65 | 81 | 14.77±0.39 | 100 | 12.29±0.57 | 83 |
| Tryptophan* | 17.94±1.05 | 100 | 15.78±2.95 | 88 | 24.37±0.39 | 100 | 22.23±1.37 | 91 |
| Tyrosine | 11.31±0.13 | 100 | 13.50±0.62 | 119 | 26.52±0.44 | 100 | 17.32±0.79 | 65 |
| Valine* | 26.90±0.44 | 100 | 33.20±6.01 | 123 | 16.87±2.06 | 100 | 21.80±0.87 | 129 |
| Total | 556.04** | 100 | 533.80** | 96 | 351.52** | 100 | 357.58** | 102 |

Presented data of amino acids content are mean values from 3 independent extractions ± standard deviation (SD). * Essential amino acids. **The sum of the average values for the three experiments.
